# Cytomegalovirus (CMV) Infection in Pregnancy Resulting in Neonatal Mortality Due to Congenital CMV: A Case Report

**DOI:** 10.7759/cureus.84413

**Published:** 2025-05-19

**Authors:** Saima Faraz, Umniyah Abu-nayla, Fadi Mirza, Nighat Aftab, Sofia A Malik

**Affiliations:** 1 Obstetrics and Gynecology, Latifa Women and Children Hospital, Dubai, ARE; 2 Obstetrics and Gynecology, Dubai Academic Health Corporation, Dubai, ARE; 3 Obstetrics and Gynecology, Dubai Medical College, Dubai, ARE

**Keywords:** congenital cytomegalovirus (ccmv), estimated fetal weight (efw), intrauterine growth restriction (iugr), pcr testing, preterm premature rupture of membranes (pprom)

## Abstract

Congenital cytomegalovirus (cCMV) infection is a major cause of neonatal morbidity that can be easily overlooked, as only a few cases are symptomatic, leading to severe complications such as hearing loss, developmental delays, and brain damage. It is one of the most common congenital infections. A 37-year-old woman with a high-risk pregnancy, including preeclampsia and type 2 diabetes, presented at 30+2 weeks gestation with abdominal pain and reduced fetal movements. Ultrasound revealed intrauterine growth restriction (IUGR) and abnormal Doppler findings, prompting an emergency cesarean section. The newborn, weighing 1100g, showed signs of CMV infection, including jaundice and hepatosplenomegaly, which was confirmed by polymerase chain reaction (PCR) testing. Despite antiviral treatment, the infant’s condition deteriorated with progressive neurological issues, such as intracranial hemorrhage and hydrocephalus, ultimately leading to death on day 39. This case highlights the severe consequences of undiagnosed CMV infection in high-risk pregnancies. It emphasizes the importance of early intervention and routine screening for maternal CMV infection in such cases.

## Introduction

Maternal-fetal cytomegalovirus (CMV) infection is the leading infectious cause of congenital malformations, neurodevelopmental impairment, and hearing loss [1], affecting approximately 1 in 200 live births globally [2]. It crosses the placenta, infecting the embryo or fetus, and is linked to adverse pregnancy outcomes such as intrauterine growth restriction (IUGR) and preterm birth, often resulting from placental pathology [3]. This case illustrates a severe manifestation of congenital CMV (cCMV) infection in a high-risk pregnancy that was initially followed up in a private healthcare setting and presented at our institution during a critical stage. The presence of IUGR and abnormal Doppler findings was compounded by the delayed recognition of maternal CMV infection. This highlights the importance of heightened clinical suspicion and routine screening for CMV, especially in high-risk pregnancies.

## Case presentation

A 37-year-old Indian woman, in her second pregnancy, presented at 30+2 weeks gestation with reduced fetal movements and lower abdominal pain. She was unbooked, having received antenatal care at a private clinic and reported to this hospital for the first time. Her medical history included preeclampsia, type 2 diabetes, and IUGR in the current pregnancy. The patient had a previous cesarean section due to preterm premature rupture of membranes (PPROM) eight years ago, delivering a healthy child.

**Figure 1:**
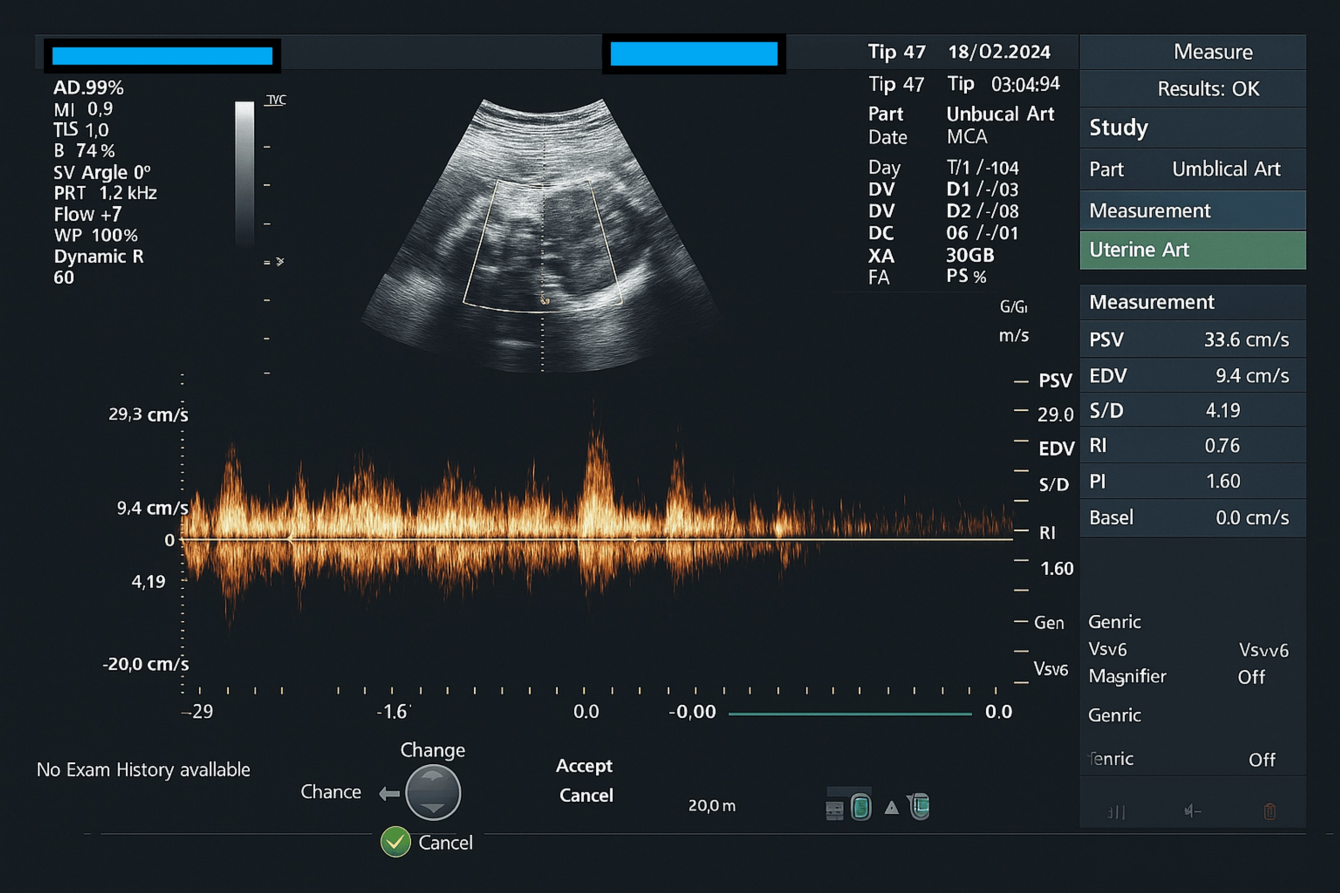
Abnormal Doppler of the middle cerebral artery of the fetus infected with CMV CMV: Cytomegalovirus

On examination, the patient was stable but appeared anxious. Ultrasound revealed breech presentation, an estimated fetal weight (EFW) of 1kg (third percentile), oligohydramnios (amniotic fluid index of 6cm), and abnormal Doppler findings, including reverse umbilical artery Doppler and abnormal middle cerebral artery Doppler (Figure 1). These findings, along with the deteriorating maternal condition, led to the transfer to our hospital. Betamethasone had already been administered for fetal lung maturity.

An emergency cesarean section was performed under spinal anesthesia due to pathological fetal heart rate patterns. A female infant weighing 1100g was delivered, with an appearance, pulse, grimace, activity, and respiration (APGAR) score of 6 at 1 minute and 8 at 5 minutes. The neonate exhibited signs of congenital infection, including jaundice, hepatosplenomegaly, petechiae, and reduced respiratory effort, requiring intubation. Histopathology of the placenta revealed chorionic villitis, suggesting viral infection, particularly CMV. Tests for rubella, syphilis, and hepatitis were negative.

The CMV polymerase chain reaction (PCR) testing of both blood and urine confirmed congenital CMV infection. The infant began treatment with ganciclovir on day 3 of life, under the supervision of an infectious disease specialist. However, by day 16, her condition worsened, with anuria, hyponatremia, hypotension, metabolic acidosis, and various deranged laboratory parameters, as shown in Table 1. Empirical antibiotics (meropenem and teicoplanin) were initiated as per protocol, and all cultures remained negative. Platelet count remained critically low, necessitating multiple transfusions.

**Table 1:** Various chemical parameters of the infant that were recorded during admission

Lab parameter	Normal range	Day 1	Day 4	Day 6	Day 8	Day 18
WBC count	5 -19 x 10^3^ /uL	0.7	1.1	1.2	1.6	1.9
RBC count	3-5.40 10^6^ / uL	4.39	3.43	4.76	4.09	3.51
Hemoglobin	11.5-16.5 g/dL	9.9	13.7	12.8	11.8	10
Platelets	100-400 x 10^2^/dL	57	18	20	35	68
C-reactive protein	< 5 mg/dL	18.3	27.7	50.4	43	19.3
Creatinine	0.20-0.40 mg/dL	1.13	0.80	0.78	0.67	0.21
Prothrombin time	13.5-16.4 sec	16	17.3	15.6	13.3	13.1
International normalised ratio	1.05-1.35	1.29	1.42	1.25	1.02	1.00
Lactate	0.5-1.6 mmol/L	2.1	1.9	2.0	1.8	1.7

A brain ultrasound on day 4 revealed grade 1 intraventricular hemorrhage (IVH), which progressed to grade 4 by day 10. The infant also developed bilateral periventricular leukomalacia and porencephalic cysts. Hydrocephalus progressed, with persistent dilation of the lateral and third ventricles.

Despite intensive care in the neonatal unit, the infant’s clinical status continued to deteriorate. On day 21, disseminated intravascular coagulation (DIC) developed, leading to pulmonary hemorrhage. Multiple resuscitation attempts, including saline boluses, platelet transfusions, and cryoprecipitate, were made. High-frequency oscillatory ventilation (HFOV) was initiated but failed to improve the clinical condition. By day 39, the infant experienced cardiac arrest. Despite resuscitation efforts, including chest compressions and adrenaline, the infant was declared deceased after no spontaneous circulation was restored.

## Discussion

Human CMV is a widespread virus that infects most individuals at some stage in their lives. It belongs to the *Herpesviridae* family, which includes other viruses such as herpes simplex virus types 1 and 2, varicella zoster virus, Epstein-Barr virus, and Kaposi’s sarcoma-associated herpesvirus (HHV-8) [4]. Cytomegalovirus infections are typically clinically silent, but about 10% to 15% of congenital infections can present with symptoms, including IUGR, petechial rash, microcephaly, intracranial calcifications, chorioretinitis, thrombocytopenia, neutropenia, and hepatosplenomegaly with associated hyperbilirubinemia and transaminitis [5].

A CMV infection during pregnancy can arise as a primary infection or from non-primary events, such as viral reactivation or reinfection. Both types are capable of transmitting the virus vertically to the fetus and may lead to similar clinical outcomes in the newborn. Following a primary maternal infection, the estimated risk of transplacental CMV transmission is about 30% to 35%, whereas non-primary infections carry a significantly lower transmission risk, approximately 1% to 3% [6]. In developed countries, the CMV seroprevalence is low, and approximately 50% of congenital CMV cases are linked to non-primary maternal infections acquired during gestation [7]. Most maternal CMV infections are asymptomatic or manifest with vague symptoms like low-grade fever, fatigue, muscle aches, and flu-like signs, occurring in about one-third of cases. Laboratory findings may reveal lymphocytosis greater than 40% and increased liver transaminases in roughly 50% of affected individuals [8].

In utero, CMV infection can significantly alter brain development, especially in neural stem cells, which are highly abundant and susceptible to the virus. Neurological injury in the fetus may arise from unchecked viral replication, cytotoxic effects of CD-8+ lymphocytes, and, when accompanied by placental insufficiency, resultant fetal hypoxia [9]. This highlights the importance of early diagnosis and management in preventing severe neurological damage [10].

Prenatal diagnostic evaluation is initiated when maternal primary CMV infection is suspected, based on clinical presentation or serological screening, or when prenatal ultrasonography reveals findings suggestive of fetal infection [11]. Postnatal confirmation of congenital CMV infection relies on virological detection of the virus in body fluids such as urine or saliva, collected within the first two to four weeks of life [12]. Clinical manifestations of cCMV infection encompass a spectrum of signs, including petechiae in approximately 76% of cases, jaundice in 67%, hepatosplenomegaly in 60%, microcephaly in 53%, IUGR in 50%, and chorioretinitis or optic atrophy in 20% of affected neonates [13]. Additionally, purpura is observed in 13% of affected neonates, while seizures are reported in about 7% [13]. Symptomatic cCMV infection is seen in approximately 10% of infected infants, and these infants may experience substantial complications, including sensorineural hearing loss, mental retardation, and even mortality in up to 30% of cases [14].

Intraventricular hemorrhage, although rare in cCMV infection, has been reported in very preterm neonates or in association with thrombocytopenia [15]. The pathogenesis of periventricular cysts observed in cCMV remains unknown [16]. A CMV-induced brain injury is thought to be neurotropic, involving direct damage to neurons. This is particularly detrimental when infection occurs in early pregnancy, potentially leading to structural brain abnormalities such as cortical dysplasia and hypoplasia of the hippocampus and cerebellum [17].

Therapeutic management of infants with cCMV infection is guided by the presence and severity of clinical manifestations. Antiviral therapy is strongly recommended for neonates presenting with severe symptoms, particularly those involving the central nervous system [18]. The primary pharmacologic agents used are intravenous ganciclovir and its oral prodrug, valganciclovir, both of which inhibit CMV replication by targeting viral DNA synthesis. Comparative studies have demonstrated that oral valganciclovir offers efficacy comparable to intravenous ganciclovir [19]. Furthermore, a phase III clinical trial has shown that intravenous ganciclovir therapy initiated in symptomatic neonates with neurological involvement leads to improved auditory outcomes and reduced neurodevelopmental impairment at a follow-up between six months and one year of age [20].

## Conclusions

This case unequivocally demonstrates the severe neonatal complications and high morbidity associated with cCMV infection. The rapid progression of intraventricular hemorrhage from grade 1 to 4 strongly supports the detrimental impact of cCMV on the neonate's health. These findings underscore the potential for significant neurological sequelae and highlight the critical need for further investigation into effective strategies for early detection, intervention, and management of cCMV to mitigate such devastating outcomes. This case also highlights the vital importance of early diagnosis, prompt intervention, and effective treatment in managing cCMV infections. 
